# An exploration of the (3+1)-dimensional negative order KdV-CBS model: Wave solutions, Bäcklund transformation, and complexiton dynamics

**DOI:** 10.1371/journal.pone.0296978

**Published:** 2024-04-16

**Authors:** Miguel Vivas-Cortez, Beenish Rani, Nauman Raza, Ghada Ali Basendwah, Mudassar Imran

**Affiliations:** 1 School of Physical and Mathematical Sciences, Faculty of Exact and Natural Sciences, Pontificia Universidad Catolica del Ecuador, Quito, Ecuador; 2 Department of Mathematics, University of the Punjab, Quaid-e-Azam Campus, Lahore, Pakistan; 3 Department of Mathematics, Near East University, TRNC, Nicosia, Turkey; 4 Department of Mathematics, King Abdulaziz University, Jeddah, Saudi Arabia; 5 College of Humanities and Sciences, Ajman University, Ajman, United Arab Emirates; College of Mathematics and Systems Science, Shandong University of Science and Technology, CHINA

## Abstract

This research paper focuses on the study of the (3+1)-dimensional negative order KdV-Calogero-Bogoyavlenskii-Schiff (KdV-CBS) equation, an important nonlinear partial differential equation in oceanography. The primary objective is to explore various solution techniques and analyze their graphical representations. Initially, two wave, three wave, and multi-wave solutions of the negative order KdV CBS equation are derived using its bilinear form. This analysis shed light on the behavior and characteristics of the equation’s wave solutions. Furthermore, a bilinear Bäcklund transform is employed by utilizing the Hirota bilinear form. This transformation yields exponential and rational function solutions, contributing to a more comprehensive understanding of the equation. The resulting solutions are accompanied by graphical representations, providing visual insights into their structures. Moreover, the extended transformed rational function method is applied to obtain complexiton solutions. This approach, executed through the bilinear form, facilitated the discovery of additional solutions with intriguing properties. The graphical representations, spanning 2D, 3D, and contour plots, serve as valuable visual aids for understanding the complex dynamics and behaviors exhibited by the equation’s solutions.

## Introduction

Partial differential equations (PDEs) have proven to be valuable tools in tackling a wide array of problems in mathematics and physics. With its foundation in various scientific disciplines, the concept of PDEs, encompassing both linear and nonlinear forms, represents one of the most advanced and dynamic areas of modern mathematics. Numerous scientific and engineering fields heavily rely on PDEs for their analytical needs. Solving PDEs is crucial in understanding and studying nonlinear physical phenomena. Their utilization has seen a significant rise in domains such as engineering [1], complex geometries [2], the processing of images [3] and finance [4]. The exploration of a diverse range of nonlinear partial differential equations (NLPDEs) is necessary for mathematically modeling complex phenomena that evolve over time. Researchers have developed various methodologies, including numerical and analytical techniques, as well as computational algorithms, to effectively solve PDEs and gain insights into complex phenomena [5]. Some of these techniques include, Homotopy analysis [6], extended generalized Kudryashov method [7], Laplace-Adomian decomposition method [8], Riccati projective equation method [9], Hirota bilinear form [10], Bäcklund transformation [11], and many others [12–14].

In recent decades, there has been significant interest and fascination in investigating the outcomes pertaining to the aforementioned factors, along with exploring the implications of successfully establishing closed-form wave solutions for a broader range of nonlinear equations. Solitons, characterized as nonlinear diffusive solutions to partial differential equations, offer a comprehensive description of various physical systems. These solitary waves, often referred to as spiral wave bundles, possess a remarkable ability to maintain their shape while moving at a constant velocity [15]. The unique particle-like characteristics displayed by solitons during propagation make them an ideal testing ground for studying nonlinear wave interactions. To uncover soliton solutions, diverse approaches have been developed. Researchers have employed various mathematical techniques, such as Raza et al. [16] employed the unified techniques to find solition solutions, Zhao [17] utilized the Kadomtsev-Petviashvili hierarchy reduction method to retrieve dark soliton solutions, Hirota bilinear and Darboux transformation(DT) methods were applied by Gao et al. [18] and Hang Yin and Lü [19] respectively, to find soliton solutions, Cheng et al. [20] employed auto-Bäcklund transformations, and Shen et al. [21] used DT to obtain multi-pole solitons. Moreover, several alternative techniques have been investigated in the literature [22–24].

This paper examines the negative order KdV-CBS model [25] given as:

$$\begin{array}{c} \psi_{xt}+\psi_{xxxy}+4\psi_{x}\psi_{xy}+2\psi_{xx}\psi_{y}+\alpha \psi_{xx}+\beta \psi_{xy}+\gamma \psi_{xz}=0. \end{array}$$ (1)

Eq (1) represents a mathematical relationship involving unspecified coefficients *α*, *β* and *γ*. This equation combines aspects of the Korteweg-de Vries (KdV) equation and the Calogero-Bogoyavlenskii-Schiff (CBS) equation.

- For *β* = *γ* = 0, Eq (1) will be reduced to negative-order KdV equation.
- For *α* = *γ* = 0, Eq (1) will be simplified to negative-order CBS equation.

The KdV equation is widely acknowledged for its complete integrability. Consequently, it offers multiple soliton solutions and showcases an extensive array of energy conservation laws. Furthermore, the interaction between the long propagating wave along the x-axis and the Riemann propagating wave along the y-axis is described by the nonlinear CBS equation.

Taking the following transformation,

$$\begin{array}{c} \psi \left(x,y,z,t\right)=2{\left(\text{ln}\;m\right)}_{x}, \end{array}$$ (2)

then, the bilinear form of Eq (1) is given by,

$$\begin{array}{c} (D_{x}D_{t}+D_{x}^{3}D_{y}+\alpha D_{x}^{2}+\beta D_{x}D_{y}+\gamma D_{x}D_{z})m\cdot m=0, \end{array}$$ (3)

where the Hirota bilinear operator is defined as,

$$\begin{array}{cc} D_{x}^{r_{1}}D_{y}^{r_{2}}D_{z}^{r_{3}}D_{t}^{r_{4}}\left(M\cdot N\right) & =(\frac{\partial }{\partial x}-\frac{\partial }{\partial x_{i}}{)}^{r_{1}}(\frac{\partial }{\partial y}-\frac{\partial }{\partial y_{i}}{)}^{r_{2}}(\frac{\partial }{\partial z}-\frac{\partial }{\partial z_{i}}{)}^{r_{3}}(\frac{\partial }{\partial t}-\frac{\partial }{\partial t_{i}}{)}^{r_{4}}\\ & \;\times M\left(x,y,z,t\right)N\left(x_{0},y_{0},z_{0},t_{0}\right){|}_{x_{i}=x,y_{i}=y,z_{i}=z,t_{i}=t}. \end{array}$$

Wazwaz [25] studied the painlevé integrability and employed the simplified Hirota method to derive N-soliton solutions of Eq (1). Also, Gandarias and Raza [26] determined the conservation laws of the above equation with the help of the multiplier method. Recently, Raza et al. [27] analyzed the interactive behavior of different wave structures for Eq (1) and also applied the modified form of the simple equation method to obtain soliton solutions.

We aim to examine the negative order KdV-CBS model using its bilinear representation. The Bäcklund transformation is a valuable mathematical technique employed in nonlinear partial differential equations, enabling the creation of novel solutions based on existing ones through a distinct relationship [28]. This transformation has broad applications in fields like soliton theory, integrable systems, and mathematical physics, facilitating the investigation of diverse solution structures and offering unique insights into the dynamics of nonlinear systems. The Bäcklund transformation has been successfully applied to various equations, including the Kadomtsev-Petviashvili equation [29], the nonlinear Schrödinger equation [30], and Supersymmetric Two-Boson equation [31]. Its versatility and effectiveness in generating new solutions have made it a valuable tool in the study of nonlinear phenomena across different disciplines.

In addition to exploring the Bäcklund transformation and its solutions, we also investigate the extended transformed rational function method to obtain complexiton solutions in the present study. This method complements the Bäcklund transformation by offering an alternative approach to tackle nonlinear partial differential equations. By employing rational function solutions and extended transform techniques, we can effectively capture the intricate dynamics of these equations and derive analytical solutions that exhibit complex behaviors [32, 33]. In this study, we will utilize the bilinear form of the given model to explore various ansatz and apply the Bäcklund transformation to derive different solutions in the form of traveling waves. Additionally, we will employ the extended transformed rational function method to obtain complexiton solutions. It is worth noting that these approaches have not been previously utilized in the existing literature for the considered model.

The paper is structured as follows: Following an introductory section, the next section delves into various wave structures and examines their dynamic behavior. Then, the bilinear Bäcklund transform is presented, uncovering exponential and rational function solutions. In the subsequent section, the extended transformed rational function algorithm is explained, and solutions are derived using this approach. Finally, a concluding summary of the paper is presented.

## Wave solutions: Three, two, and multiwave analysis

In this section, we have explored various test functions to illustrate different types of wave solutions: two-wave, three-wave, and multi-wave. We have also provided visual representations to enhance understanding.

From the bilinear form (3), we have,

$$\begin{array}{c} mm_{xt}-m_{x}m_{t}+mm_{xxxy}+3m_{xy}m_{xx}-3m_{xxy}m_{x}-m_{xxx}m_{y}+\alpha \left(mm_{xx}-m_{x}^{2}\right)\\ +\beta \left(mm_{xy}-m_{x}m_{y}\right)+\gamma \left(mm_{xz}-m_{x}m_{z}\right)=0. \end{array}$$ (4)

### Two-wave solutions

The function that is employed for obtaining two-wave solutions to Eq (1) is presented as follows:

$$\begin{array}{c} m\left(x,y,z,t\right)=g_{1}e^{-\theta }+g_{2}e^{\theta }+g_{3}\;\text{sin}\;\left(\varphi \right)+g_{4}\;\text{sinh}\left(\chi \right), \end{array}$$ (5)

where, *θ* = *a*_1_*y* + *p*_1_*t* + *q*_1_*z* + *r*_1_*x*, *φ* = *a*_2_*y* + *p*_2_*t* + *q*_2_*z* + *r*_2_*x* and *χ* = *a*_3_*y* + *p*_3_*t* + *q*_3_*z* + *r*_3_*x*. Through the substitution of Eq (5) into Eq (4) and the subsequent equating of coefficients to zero, the following expression is obtained:

$$\begin{array}{cc} & g_{4}=\frac{\sqrt{2}\sqrt{g_{3}^{2}\left(a_{3}+p_{3}\right)}}{\sqrt{4a_{3}+p_{3}}},\;g_{2}=\alpha =g_{1}=0,\;a_{2}=\frac{p_{3}}{2},\;p_{2}=-2a_{3}, \end{array}$$ (6)

putting Eq (6) in Eq (5), yields,

$$\begin{array}{cc} & m\left(x,y,z,t\right)=\frac{\sqrt{2}\sqrt{g_{3}^{2}\left(a_{3}+p_{3}\right)}\text{sinh}\left(a_{3}y+p_{3}t+q_{3}z+r_{3}x\right)}{\sqrt{4a_{3}+p_{3}}}-g_{3}\;\text{sin}(2a_{3}t-\frac{p_{3}y}{2}-q_{2}z-r_{2}x). \end{array}$$ (7)

**Fig 1:**
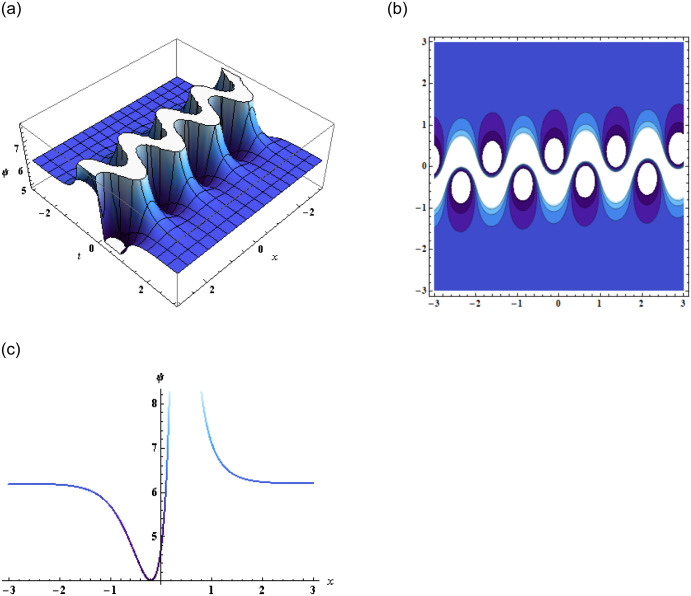
Graphical representation of Eq (8) when $g_{3}=0.1,q_{3}=2.17,z=2,a_{3}=-2.1,q_{2}=1.1,y=2,p_{3}=\frac{-1}{7},r_{2}=-\frac{0.1}{5},r_{3}=3.1$.

Thus, substituting Eq (7) in Eq (2), we get the solution of Eq (1), which is then represented in Fig 1.

$$\begin{array}{c} \psi \left(x,y,z,t\right)=\frac{2(r_{2}g_{3}\;\text{cos}\;(2a_{3}t-\frac{p_{3}y}{2}-q_{2}z-r_{2}x)+\frac{\sqrt{2}r_{3}\sqrt{g_{3}^{2}\left(a_{3}+p_{3}\right)}\;\text{cosh}\left(a_{3}y+p_{3}t+q_{3}z+r_{3}x\right)}{\sqrt{4a_{3}+p_{3}}})}{\frac{\sqrt{2}\sqrt{g_{3}^{2}\left(a_{3}+p_{3}\right)}\;\text{sinh}\left(a_{3}y+p_{3}t+q_{3}z+r_{3}x\right)}{\sqrt{4a_{3}+p_{3}}}-g_{3}\;\text{sin}\;(2a_{3}t-\frac{p_{3}y}{2}-q_{2}z-r_{2}x)}. \end{array}$$ (8)

### Three-wave solutions

The function employed for obtaining three-wave solutions to Eq (1) is presented as follows:

$$\begin{array}{c} m\left(x,y,z,t\right)=g_{1}e^{\theta }+e^{-\theta }+g_{2}\;\text{cos}\left(\chi \right)+g_{3}\;\text{sin}\;\left(\phi \right), \end{array}$$ (9)

where, *θ* = (*x* + *p*_1_*y* + *q*_1_*z* + *r*_1_*t*), *χ* = *x* + *p*_2_*y* + *q*_2_*z* + *r*_2_*t* and *φ* = *x* + *p*_3_*y* + *q*_3_*z* + *r*_3_*t*. By substituting Eq (9) into Eq (4) and setting the coefficients to zero, the following set of solution is obtained:

$$\begin{array}{c} g_{1}=g_{2}=0,\;p_{1}=-2p_{3},\;r_{1}=-\alpha +2p_{3}(\alpha \beta +1)-\alpha \gamma q_{1},\\ \;r_{3}=-\alpha -p_{3}(\alpha \beta -4)-\alpha \gamma q_{3}. \end{array}$$ (10)

plugging Eq (10) into Eq (9), gives,

$$\begin{array}{cc} & m\left(x,y,z,t\right)=\text{exp}(-t(-\alpha +2p_{3}(\alpha \beta +1)-\alpha \gamma q_{1})+2p_{3}y-q_{1}z-x)\\ & +g_{3}\;\text{sin}\;(t(-\alpha -p_{3}(\alpha \beta -4)-\alpha \gamma q_{3})+p_{3}y+q_{3}z+x). \end{array}$$ (11)

**Fig 2:**
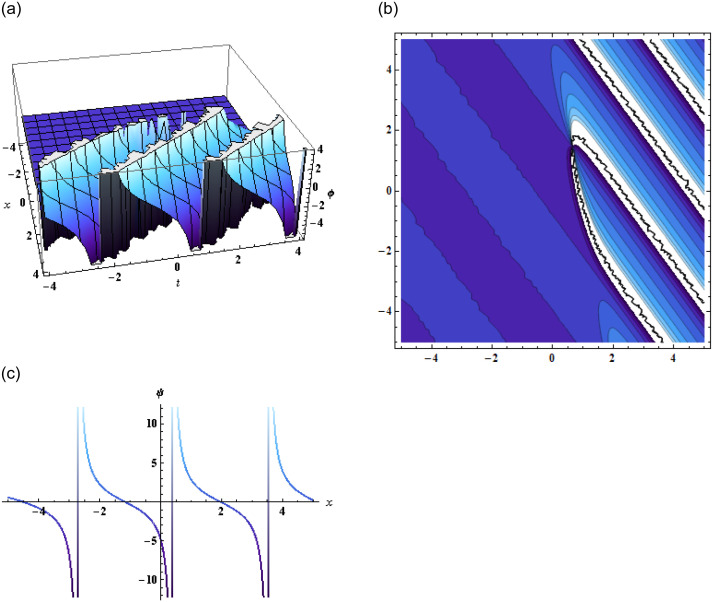
Graphical representation of Eq (1) using Eq (11) when $g_{3}=-0.01,q_{3}=-0.17,z=0,\alpha =-2.2,y-=0,p_{3}=\frac{-1}{7},\beta =2,\alpha =-1,\gamma =-1,q_{1}=-1$.

Inserting the above expression in Eq (2), we get the solution of Eq (1), which is then represented in Fig 2.

### Multi-wave solutions

The function employed for obtaining multi-wave solutions to Eq (1) is presented as follows:

$$\begin{array}{c} m\left(x,y,z,t\right)=g_{1}\;\text{cosh}\left(\chi \right)+g_{2}\;\text{cos}\left(\theta \right)+g_{3}\;\text{cosh}\left(\phi \right), \end{array}$$ (12)

where, *θ* = *x* + *a*_1_*y* + *q*_1_*z* + *p*_1_*t*, *χ* = *x* + *a*_2_*y* + *q*_2_*z* + *p*_2_*t* and *φ* = *x* + *a*_3_*y* + *q*_3_*z* + *p*_3_*t*. By substituting Eq (12) into Eq (4) and setting the coefficients to zero, the following set of solution is obtained:

$$\begin{array}{cc} & g_{3}=0,\;a_{1}=\frac{-2a_{2}g_{1}^{2}-a_{2}g_{2}^{2}}{g_{2}^{2}},\;p_{2}=\alpha -a_{2}\alpha \beta -q_{2}\alpha \gamma ,\\ & p_{1}=\frac{\left(-4a_{2}g_{1}^{2}-4a_{2}g_{2}^{2}-g_{2}^{2}\alpha +2a_{2}g_{1}^{2}\alpha \beta +a_{2}g_{2}^{2}\alpha \beta -q_{1}g_{2}^{2}\alpha \gamma \right)}{g_{2}^{2}}. \end{array}$$ (13)

Substituting Eq (13) in Eq (12), gives,

$$\begin{array}{cc} & m\left(x,y,z,t\right)=[g_{2}\;\text{cos}[x+\frac{(-2a_{2}g_{1}^{2}-a_{2}g_{2}^{2})y}{g_{2}^{2}}+q_{1}z\\ & +\frac{t(-4a_{2}g_{1}^{2}-4a_{2}g_{2}^{2}-g_{2}^{2}\alpha +2a_{2}g_{1}^{2}\alpha \beta +a_{2}g_{2}^{2}\alpha \beta -q_{1}g_{2}^{2}\alpha \gamma )}{g_{2}^{2}}]\\ & +g_{1}\;\text{cosh}\left[x+a_{2}y+q_{2}z+t\left(-\alpha -a_{2}\alpha \beta -q_{2}\alpha \gamma \right)\right]. \end{array}$$ (14)

**Fig 3:**
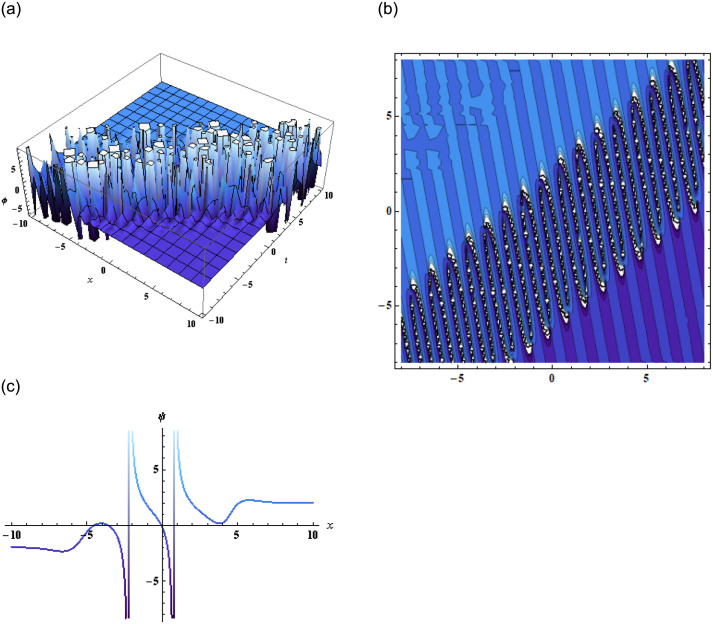
Graphical representation of Eq (1) using Eq (14) when $g_{1}=0.1,q_{1}=-1,z=2,g_{2}=3.8,q_{2}=1.1,\alpha =-2.2,y=2,a_{1}=\frac{1}{9},a_{2}=\frac{-1}{7},\beta =2,\alpha =-1,\gamma =-1$.

Plugging this expression in Eq (2), we get the solution of Eq (1), as shown in Fig 3.

## Bilinear Bäcklund transformation

To find the bilinear Bäcklund transformation by using the bilinear form given in Eq (3), we take into consideration another function *n*(*x*, *y*, *z*, *t*) as the bilinear form’s solution that is,

$$\begin{array}{c} (D_{x}D_{t}+D_{x}^{3}D_{y}+\alpha D_{x}^{2}+\beta D_{x}D_{y}+\gamma D_{x}D_{z})n\cdot n=0, \end{array}$$ (15)

We consider the following expression by utilizing [34],

$$\begin{array}{c} \begin{array}{cc} P= & \left[(D_{x}D_{t}+D_{x}^{3}D_{y}+\alpha D_{x}^{2}+\beta D_{x}D_{y}+\gamma D_{x}D_{z})n\cdot n\right]m^{2}\\ & -n^{2}\left[(D_{x}D_{t}+D_{x}^{3}D_{y}+\alpha D_{x}^{2}+\beta D_{x}D_{y}+\gamma D_{x}D_{z})m\cdot m\right]. \end{array} \end{array}$$ (16)

By employing the following properties of the Hirota Bilinear operator [35] in the above equation,

$$\begin{array}{cc} & (D_{x}D_{t}v\cdot v)w^{2}-v^{2}(D_{x}D_{t}w\cdot w)=2D_{x}(D_{t}v\cdot w)\cdot wv,\\ & 2(D_{x}^{3}D_{y}v\cdot v)w^{2}-2(D_{x}^{3}D_{y}w\cdot w)v^{2}=D_{x}[\left(3D_{x}^{2}D_{y}v\cdot w\right)\cdot wv+\left(3D_{x}^{2}v\cdot w\right)\cdot \left(D_{y}w\cdot v\right)\\ & +\left(6D_{x}D_{y}v\cdot w\right)\cdot \left(D_{x}w\cdot v\right)]+D_{y}[\left(D_{x}^{3}v\cdot w\right)\cdot wv+\left(3D_{x}^{2}v\cdot w\right)\cdot \left(D_{x}w\cdot v\right),\\ & (D_{x}D_{y}v\cdot v)w^{2}-v^{2}(D_{x}D_{y}w\cdot w)=2D_{x}(D_{y}v\cdot w)\cdot wv,\\ & (D_{x}^{2}v\cdot v)w^{2}-v^{2}(D_{x}^{2}w\cdot w)=2D_{x}(D_{x}v\cdot w)\cdot wv,\\ & (D_{d}(D_{e}v.w).wv=D_{e}(D_{d}v.w).wv. \end{array}$$

Thus, Eq (16) is transformed into,

$$\begin{array}{cc} & 2P=\left[(D_{x}D_{t}+D_{x}^{3}D_{y}+\alpha D_{x}^{2}+\beta D_{x}D_{y}+\gamma D_{x}D_{z})n\cdot n\right]m^{2}\\ & \;\;\;\;\;-n^{2}\left[(D_{x}D_{t}+D_{x}^{3}D_{y}+\alpha D_{x}^{2}+\beta D_{x}D_{y}+\gamma D_{x}D_{z})m\cdot m\right]\\ & \;\;=\left(D_{t}D_{x}n\cdot n\right)m^{2}-n^{2}\left(D_{t}D_{x}m\cdot m\right)+[\left(D_{x}^{3}D_{y}n\cdot n\right)m^{2}-n^{2}\left(D_{x}^{3}D_{y}m\cdot m\right)]\\ & \;\;\;\;\;+\alpha [[\left(D_{x}^{2}n\cdot n\right)m^{2}-n^{2}\left(D_{x}^{2}m\cdot m\right)]+\beta \left[\left(D_{x}D_{y}n\cdot n\right)m^{2}-n^{2}\left(D_{x}D_{y}m\cdot m\right)\right]\\ & \;\;\;\;\;+\gamma [\left(D_{x}D_{z}n\cdot n\right)m^{2}-n^{2}\left(D_{x}D_{z}m\cdot m\right)]\\ & \;\;\;=4D_{x}(D_{t}n\cdot m)\cdot mn+D_{x}[\left(3D_{x}^{2}D_{y}n\cdot m\right)\cdot mn+\left(3D_{x}^{2}n\cdot m\right)\cdot \left(D_{y}m\cdot n\right)\\ & \;\;\;\;\;+\left(6D_{x}D_{y}n\cdot m\right)\cdot \left(D_{x}m\cdot n\right)]+D_{y}[\left(D_{x}^{3}n\cdot m\right)\cdot mn+\left(3D_{x}^{2}n\cdot m\right)\cdot \left(D_{x}m\cdot n\right)\\ & \;\;\;\;\;+4\alpha [[D_{x}\left(D_{x}n\cdot m\right)\cdot mn]+4\beta [[D_{x}\left(D_{y}n\cdot m\right)\cdot mn]+4\gamma [[D_{x}\left(D_{z}n\cdot m\right)\cdot mn]\\ & \;\;\;=D_{x}[(3D_{x}^{2}D_{y}+\upsilon_{1}D_{y}+4\alpha D_{x}+4D_{t}+4\gamma D_{z}+\upsilon_{2})n\cdot m]\cdot mn\\ & \;\;\;\;\;+D_{x}[(3D_{x}^{2}+\upsilon_{4}D_{y}+\upsilon_{6})n\cdot m]\cdot (D_{y}m\cdot n)+D_{x}[(6D_{x}D_{y}+6\upsilon_{7}D_{x})n\cdot m]\cdot (D_{x}m\cdot n)\\ & \;\;\;\;\;+D_{y}[(D_{x}^{3}-\upsilon_{1}D_{x}+4\beta D_{x}+\upsilon_{3})n\cdot m]\cdot mn\\ & \;\;\;\;\;+D_{y}[(3D_{x}^{2}+\upsilon_{5}D_{x}-\upsilon_{6})n\cdot m]\cdot (D_{x}m\cdot n), \end{array}$$

Wherein seven arbitrary parameters have been introduced. The reason for the coefficients of *υ*_*i*_, (*i* = 1, 2, 3…7) to be zero in the aforementioned deduction is due to the property of the Hirota bilinear operator *D*_*a*_*n* ⋅ *n* = 0, *D*_*x*_*n* ⋅ *m* = −*D*_*x*_*m* ⋅ *n*, *D*_*x*_(*D*_*y*_*n* ⋅ *m*)⋅*mn* = *D*_*y*_(*D*_*x*_*n* ⋅ *m*)⋅*mn*. As a result, the expression representing the Bäcklund transform of Eq (1) can be expressed as,

$$\begin{array}{c} \begin{array}{cc} & (3D_{x}^{2}D_{y}+\upsilon_{1}D_{y}+4\alpha D_{x}+4D_{t}+4\gamma D_{z}+\upsilon_{2})n\cdot m=0,\\ & (3D_{x}^{2}+\upsilon_{4}D_{y}+\upsilon_{6})n\cdot m=0,\\ & (6D_{x}D_{y}+6\upsilon_{7}D_{x})n\cdot m=0,\\ & (D_{x}^{3}-\upsilon_{1}D_{x}+4\beta D_{x}+\upsilon_{3})n\cdot m=0,\\ & (3D_{x}^{2}+\upsilon_{5}D_{x}-\upsilon_{6})n\cdot m=0. \end{array} \end{array}$$ (17)

We consider the solution *n* = 1 for the bilinear form (15) and Eq (1) with the solution *ψ*(*x*, *y*, *z*, *t*) = 2(ln *m*)_*x*_ = 0 for the negative order KdV-CBS equation.

Now, by using the following property,

$$\begin{array}{c} D_{d}^{e}m\cdot 1=\frac{\partial^{e}}{\partial d^{e}}m. \end{array}$$

Then, the bilinear Bäcklund transformation Eq (17) undergoes a conversion into a set of linear partial differential equations,

$$\left\{\begin{array}{l} 3m_{xxy}+\upsilon_{1}m_{y}+4\alpha m_{x}+4m_{t}+4\gamma m_{z}+\upsilon_{2}=0,\\ 3m_{xx}+\upsilon_{4}m_{y}+\upsilon_{6}=0,\\ 6m_{xy}+6\upsilon_{7}m_{x}=0,\\ m_{xxxx}-\upsilon_{1}m_{x}+4\beta m_{x}+\upsilon_{3}=0,\\ 3m_{xx}+\upsilon_{5}m_{x}-\upsilon_{6}=0. \end{array}\right.$$ (18)

### Exponential wave solution

We consider an exponential-function solution for bilinear Form Eq (3) as,

$$\begin{array}{c} \begin{array}{cc} & m(x,y,z,t)=1+\mu \;e^{jx+ky+lz+pt}, \end{array} \end{array}$$ (19)

with *j*, *k*, *l*,*p* and *μ* being the constants. Inserting above equation into Eq (18) and selecting *υ*_2_ = *υ*_6_ = 0, *υ*_3_ = 0, we get the constraints by solving Eq (18) as follows:

$$\left\{\begin{array}{l} j=j,k=k,l=l,\;p=-j^{2}k-\alpha j-\beta k-\gamma l,\;\upsilon_{1}=j^{2}+4\beta ,\\ \upsilon_{4}=-3\frac{j^{2}}{k},\;\upsilon_{5}=-3j,\;\upsilon_{7}=-k,\; \end{array}\right.$$

thus, the exponential wave solution for Eq (1) is,

$$\begin{array}{c} \psi \left(x,y,z,t\right)=2\;\frac{\mu \;e^{jx+ky+lz}j}{e^{(j^{2}k+\alpha \;j+\beta \;k+\gamma \;l)t}+\mu \;e^{jx+ky+lz}}. \end{array}$$

### Rational function solution

To retrieve the rational functional solution for bilinear Form Eq (3), we suppose a polynomial function as follows,

$$\begin{array}{c} m(x,y,z,t)=jx+ky+lz-pt, \end{array}$$

where *j*, *k*, *l*, *p* are constants to be evaluated later. Similarly selecting *υ*_2_ = *υ*_6_ = 0, *υ*_3_ = 0 and substituting above equation into Eq (18), we get,

$$\begin{array}{c} \{j=j,k=k,l=l,\;p=\alpha \;j+\gamma \;l,+\beta k,\;\upsilon_{1}=4\beta ,\;\upsilon_{4}=\upsilon_{5}=\upsilon_{7}=0, \end{array}$$

thus, the rational function solution for Eq (1) is,

$$\begin{array}{c} \psi =-2\;\frac{j}{\alpha \;jt+\gamma \;lt+\beta kt-jx-ky-lz}. \end{array}$$ (20)

**Fig 4:**
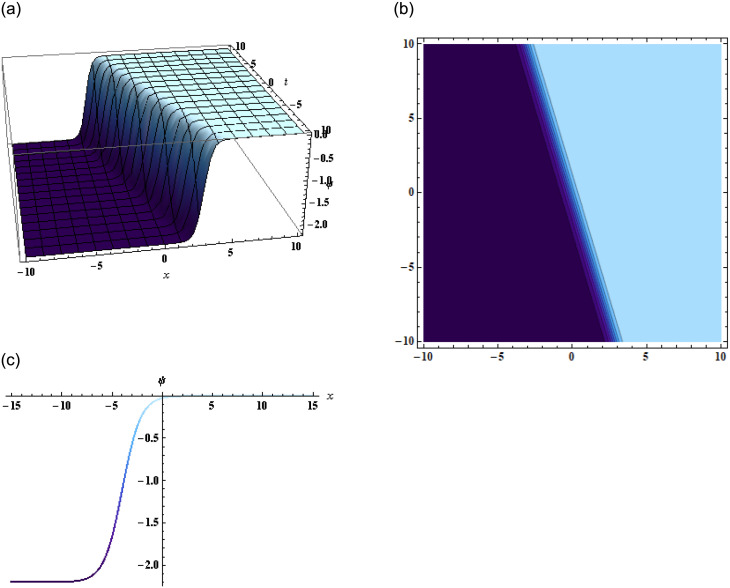
Graphical representation of exponential function solution when *α* = 0.03, *β* = 3, *γ* = 0.5, *j* = −1.1, *k* = 1, *l* = −1, *μ* = 0.5, *y* = 1, *z* = 1.

**Fig 5:**
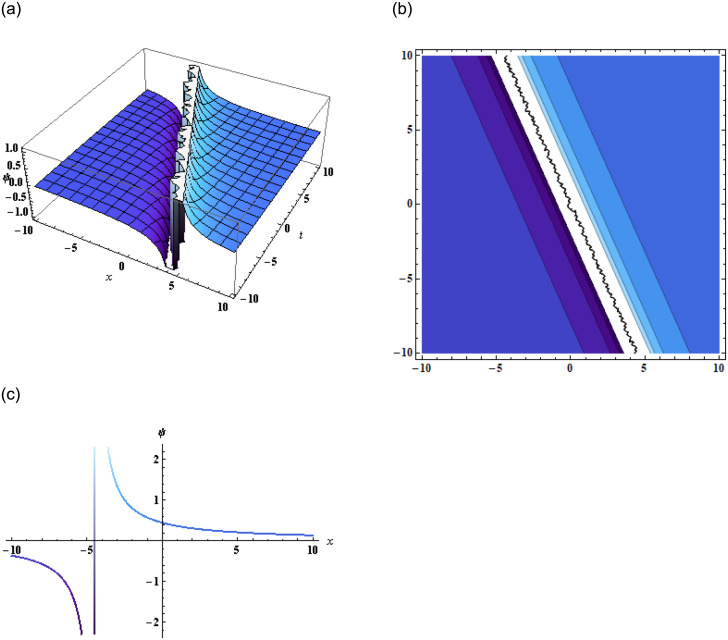
Graphical representation of rational function solution when *α* = 0.03, *β* = 3, *γ* = 0.5, *j* = −1.1, *k* = 1, *l* = −1, *y* = 1, *z* = 1.

The exponential and rational function solutions are then represented in graphs as shown in Figs 4 and 5 respectively.

## The extended transformed rational function approach

This section will cover each step of the extended transformed rational function method.

**Stage 1**: We begin with general form of a bilinear Hirota NLPDE involving the independent variables *x*, *y*, *z*, and *t*, and the dependent variable *m*. The equation is expressed as follows:

$$\begin{array}{c} \begin{array}{c} B(D_{x},D_{y},D_{z},D_{t}.....)m.m=0, \end{array} \end{array}$$ (21)

where *D*_*x*_, *D*_*y*_, *D*_*z*_, *D*_*t*_,…., represents Hirota differential operators defined by,

$$\begin{array}{c} D_{\omega }^{s}k\left(\omega \right)\cdot l\left(\omega \right)={\left(\partial_{\omega }-\partial_{\omega^{\prime }}\right)}^{s}k\left(\omega \right)l\left(\omega^{\prime }\right){|}_{\omega^{\prime }=\omega })=\partial_{\omega^{\prime }}^{s}k\left(\omega +\omega^{\prime }\right)l\left(\omega -\omega^{\prime }\right){|}_{\omega^{\prime }=0}. \end{array}$$

**Stage 2**: Next, we establish the solution of Eq (21) as follows,

$$\begin{array}{c} p=\frac{v(\varpi_{1},\varpi_{2})}{w(\varpi_{1},\varpi_{2})}, \end{array}$$ (22)

where *v*(*ϖ*_1_, *ϖ*_2_) and *w*(*ϖ*_1_, *ϖ*_2_) are polynomials and for *ϖ*_1_ and *ϖ*_2_, the following differential equations are admissible,

$$\begin{array}{c} \varpi_{1}^{\prime \prime }=\frac{d^{2}\varpi_{1}}{d\varphi_{1}^{2}}=-\varpi_{1}, \end{array}$$ (23)

$$\begin{array}{c} \varpi_{2}^{\prime \prime }=\frac{d^{2}\varpi_{2}}{d\varphi_{2}^{2}}=\varpi_{2}, \end{array}$$ (24)

here, *φ*_1_ = *a*_1_*x* + *ba*_1_*y* + *ca*_1_*z* + *n*_1_*t* + *d*, *φ*_2_ = *a*_2_*x*+ + *ba*_2_*y* + *ca*_2_*z* + *n*_2_*t* + *f*,

The values of *a*_1_, *a*_2_, *n*_1_ and *n*_2_ can be determined at a later stage, while *d* and *f* are arbitrary constants. By employing the following approach, one can readily obtain the solutions for Eqs (23) and (24):

$$\begin{array}{c} \begin{array}{c} \varpi_{1}=\pm \text{sin}\;\varphi_{1}\;\;\text{or}\;\;\varpi_{1}=\pm \text{cos}\;\varphi_{1},\\ \varpi_{2}=\pm \text{sinh}\;\varphi_{2}\;\text{or}\;\varpi_{2}=\pm \text{cosh}\;\varphi_{2}, \end{array} \end{array}$$ (25)

the equalities obtained from Eq (25) are,

$$\begin{array}{c} \varpi_{1}^{\prime 2}=1-\varpi_{1}^{2}\;\text{and}\;\varpi_{2}^{\prime 2}=1+\varpi_{2}^{2}. \end{array}$$ (26)

**Stage 3**: Then, Eq (21) can be modified into an algebraic equation that contains *a*_*i*_ and *n*_*i*_ by selecting appropriate *v*(*ϖ*_1_, *ϖ*_2_), *w*(*ϖ*_1_, *ϖ*_2_). Next, we find precise complexiton solutions to Eq (21) utilizing symbolic computation.

### Exploring solutions with the extended transformed rational function approach

In this section, we will explore the key aspects of the extended transform rational function. Suppose that,

$$\begin{array}{c} m\left(x,y,z,t\right)=K\varsigma_{1}\left(\varphi_{1}\right)+L\varsigma_{2}\left(\varphi_{2}\right), \end{array}$$ (27)

where K, L are arbitrary constants and $\varsigma_{1},\varsigma_{2}$ are functions of *φ*_1_, *φ*_2_ respectively, also,

$$\begin{array}{c} \begin{array}{cc} & \varphi_{1}=a_{1}x+ba_{1}y+ca_{1}z+n_{1}t+d,\\ & \varphi_{2}=a_{2}x++ba_{2}y+ca_{2}z+n_{2}t+f. \end{array} \end{array}$$ (28)

From the bilinear form (3), under the transformation (2), the negative order KdV-CBS equation can be written as,

$$\begin{array}{c} \begin{array}{cc} & mm_{xt}-m_{x}m_{t}+mm_{xxxy}+3m_{xy}m_{xx}-3m_{xxy}m_{x}-m_{xxx}m_{y}+\alpha \left(mm_{xx}-m_{x}^{2}\right)\\ & +\beta \left(mm_{xy}-m_{x}m_{y}\right)+\gamma \left(mm_{xz}-m_{x}m_{z}\right)=0. \end{array} \end{array}$$ (29)

Substituting Eq (27) into Eq (29) and using the relations,

$$\begin{array}{c} \begin{array}{cc} & \varsigma_{1}^{\prime \prime }=-\varsigma_{1}\;,\;\;\;\;\varsigma_{2}^{\prime \prime }=\varsigma_{2},\\ & \varsigma_{1}^{\prime 2}=1-\varsigma_{1}^{2}\;,\;\;\;\;\varsigma_{2}^{\prime 2}=1+\varsigma_{2}^{2}, \end{array} \end{array}$$ (30)

the result can be represented in polynomial form by using notations $\varsigma_{1}^{2}$, $\varsigma_{2}^{2}$, $\varsigma_{1}\varsigma_{2}$ and $\varsigma_{1}^{\prime }\varsigma_{2}^{\prime }$. Collecting the coefficients for $\varsigma_{1}^{2}$, $\varsigma_{2}^{2}$, $\varsigma_{1}\varsigma_{2}$ and $\varsigma_{1}^{\prime }\varsigma_{2}^{\prime }$ and equating them to zero we get an algebraic system of equations, as defined in Eq (31).

$$\begin{array}{c} 4\;b{a_{1}}^{3}a_{2}-4\;ba_{1}{a_{2}}^{3}-2\;b\beta \;a_{1}a_{2}-2\;c\gamma a_{1}a_{2}-2\;\alpha \;a_{1}a_{2}-a_{1}n_{2}-a_{2}n_{1}=0, \end{array}$$ (31)

$$\begin{array}{c} b{a_{1}}^{4}-6\;b{a_{1}}^{2}{a_{2}}^{2}+b{a_{2}}^{4}-b\mu \;{a_{1}}^{2}+b\beta \;{a_{2}}^{2}-c\gamma {a_{1}}^{2}+c\gamma {a_{2}}^{2}-\alpha \;{a_{1}}^{2}+\alpha \;{a_{2}}^{2}-a_{1}n_{1}+a_{2}n_{2}=0, \end{array}$$

$$\begin{array}{c} \begin{array}{cc} & 4\;K^{2}b{a_{1}}^{4}-4\;L^{2}b{a_{2}}^{4}-K^{2}b\beta \;{a_{1}}^{2}-L^{2}b\beta \;{a_{2}}^{2}-K^{2}c\gamma {a_{2}}^{2}-L^{2}c\gamma {a_{1}}^{2}-L^{2}a_{2}n_{2}-K^{2}a_{1}n_{1}\\ & -L^{2}\alpha \;{a_{2}}^{2}-K^{2}\alpha \;{a_{1}}^{2}=0. \end{array} \end{array}$$

The evaluation of the previously mentioned system of simultaneous equations, Eq (31), yields the following results:

$$\begin{array}{c} K=\frac{La_{2}}{a_{1}},\;n_{1}=-a_{1}(-b{a_{1}}^{2}+3\;b{a_{2}}^{2}+b\beta +c\gamma +\alpha ), \end{array}$$ (32)

$$\begin{array}{c} \;n_{2}=-(-3\;b{a_{1}}^{2}+b{a_{2}}^{2}+b\beta +c\gamma +\alpha )a_{2}. \end{array}$$

Therefore, by the use of Eq (32), we get the solution as,

$$\begin{array}{c} \begin{array}{cc} m(x,y,z,t) & =K[\text{sin}(a_{1}x+ba_{1}y+ca_{1}z-a_{1}(-b{a_{1}}^{2}+3\;b{a_{2}}^{2}+b\beta +c\gamma +\alpha )t+d)\\ & +\frac{a_{1}\;\text{sinh}\;(a_{2}x+ba_{2}y+ca_{2}z-(-3\;b{a_{1}}^{2}+b{a_{2}}^{2}+b\beta +c\gamma +\alpha )a_{2}t+f)}{a_{2}}]. \end{array} \end{array}$$ (33)

**Fig 6:**
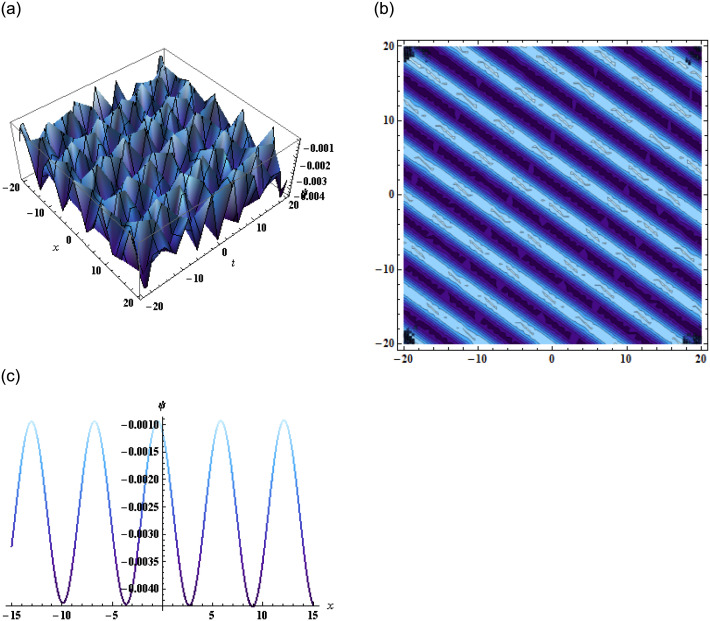
Graphical representation of Eq (1) using Eq (33) when *b* = −9, *c* = −1, *d* = −22, *f* = 1, *α* = −0.5, *β* = 3, *a*_1_ = −1, *a*_2_ = −0.001, *γ* = −0.2, *y* = 1, *z* = 2.

**Fig 7:**
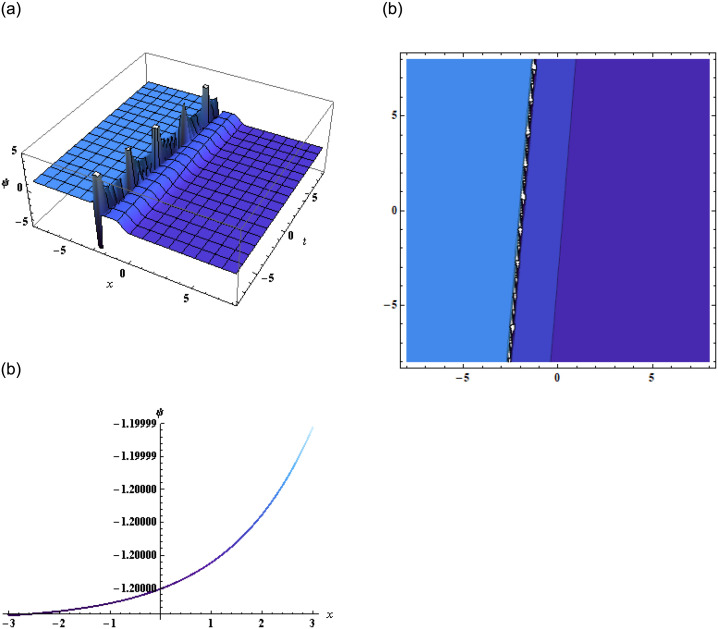
Graphical representation of Eq (1) using Eq (34) when $b=-0.9,c=-\frac{1}{1},d=-\frac{2}{7},f=\frac{3}{5},\alpha =15,\beta =3,a_{1}=-0.001,a_{2}=-0.6,\gamma =\frac{1}{5},y=10,z=12$.

Or,

$$\begin{array}{c} \begin{array}{cc} m(x,y,z,t) & =K[\text{cos}(a_{1}x+ba_{1}y+ca_{1}z-a_{1}(-b{a_{1}}^{2}+3\;b{a_{2}}^{2}+b\beta +c\gamma +\alpha )t+d)\\ & +\frac{a_{1}\;\text{sinh}\;(a_{2}x+ba_{2}y+ca_{2}z-(-3\;b{a_{1}}^{2}+b{a_{2}}^{2}+b\beta +c\gamma +\alpha )a_{2}t+f)}{a_{2}}]. \end{array} \end{array}$$ (34)

where, *a*_1_, *a*_2_, *b*, *c*, and *K* are arbitrary constants. Putting Eq (33) or Eq (34) into Eq (2) we get the solution *ψ*(*x*, *y*, *z*, *t*) which is then represented in Figs 6 and 7.

## Concluding remarks

In conclusion, this study focused on the (3+1)-dimensional negative order KdV-CBS equation and explored various solution techniques, analyzing their results through graphical representations. By employing the bilinear form of the equation, we obtained two wave, three-wave, and multi-wave solutions, which were graphically displayed in the form of 2D, 3D, and contour plots as shown in Figs 1–3. These visualizations provided valuable insights into the wave behavior and characteristics of the equation.

Additionally, the application of the bilinear Bäcklund transform using the Hirota bilinear form allowed us to derive exponential and rational function solutions. The accompanying graphical representations further enhanced our understanding of the equation’s solutions and their structures showing dynamics of the kink soliton in Fig 4 and singular kink soliton in Fig 5. Furthermore, by employing the extended transformed rational function method through the bilinear form, complexiton solutions were obtained, showcasing intriguing properties. The graphical representations of these solutions given by Figs 6 and 7, offered a vivid depiction of their complex dynamics.

The results of this study contribute to a deeper understanding of the negative order KdV-CBS equation and its solutions. The obtained wave solutions, as well as the exponential, rational function, and complexiton solutions, provide valuable insights into the intricate behavior and nature of this nonlinear equation. Understanding the solutions of the considered model and their characteristics can aid in predicting and analyzing various physical phenomena, such as wave propagation, soliton dynamics, and coherent structures. The insights gained from this study can therefore have practical applications in these domains, fostering advancements in technology and scientific research.

In summary, this research has not only expanded our knowledge of the negative order KdV-CBS equation and its solutions but has also provided a foundation for further exploration and utilization of this model in diverse scientific and technological applications.
